# Microbiome-derived cancer: the catabolism of bilirubin to urobilin in the liver–gut axis and its consequences

**DOI:** 10.1093/gastro/goag065

**Published:** 2026-07-03

**Authors:** Jayela M Davis, Annointing Ogbewekon, Darren M Gordon, Zachary A Kipp, Genesee J Martinez, Margaret A Stefater-Richards, Karl-Heinz Wagner, Terry D Hinds

**Affiliations:** Drug Disease & Discovery D3 Research Center, Department of Pharmacology and Nutritional Sciences, University of Kentucky College of Medicine, Lexington, KY 40508, United States; Markey Cancer Center, University of Kentucky, Lexington, KY 40508, United States; Drug Disease & Discovery D3 Research Center, Department of Pharmacology and Nutritional Sciences, University of Kentucky College of Medicine, Lexington, KY 40508, United States; Department of Surgery, University of Iowa Hospitals and Clinics, Iowa City, IA 52242, United States; Drug Disease & Discovery D3 Research Center, Department of Pharmacology and Nutritional Sciences, University of Kentucky College of Medicine, Lexington, KY 40508, United States; Drug Disease & Discovery D3 Research Center, Department of Pharmacology and Nutritional Sciences, University of Kentucky College of Medicine, Lexington, KY 40508, United States; Division of Endocrinology, Boston Children’s Hospital, Boston, MA 02115, United States; Harvard Medical School, Harvard University, Boston, MA 02115, United States; Department of Nutritional Sciences, University of Vienna, Vienna 1090, Austria; Drug Disease & Discovery D3 Research Center, Department of Pharmacology and Nutritional Sciences, University of Kentucky College of Medicine, Lexington, KY 40508, United States; Markey Cancer Center, University of Kentucky, Lexington, KY 40508, United States; Barnstable Brown Diabetes Center, University of Kentucky, Lexington, KY 40508, United States

**Keywords:** colon cancer, obesity, insulin resistance, gut dysbiosis, urobilinogen, BilR

## Abstract

Colorectal cancer (CRC) is the second leading cause of cancer-related deaths globally and is associated with factors, such as obesity, inflammation, and metabolic disorders. Bilirubin, a byproduct of heme degradation, is increasingly recognized as a signaling molecule with antioxidant properties that protect against obesity by reducing oxidative stress, decreasing inflammation, and activating the nuclear receptor PPARα, which enhances fat metabolism and utilization. The gut microbiome converts bilirubin to urobilinogen via bilirubin reductase, which is then rapidly oxidized to urobilin, thereby influencing colon cancer outcomes. Urobilin may contribute to CRC by being linked to insulin resistance and inflammation in obese individuals, and it could cause DNA damage. Additionally, it may serve as a biomarker for CRC, obesity, insulin-resistant diabetes, and irritable bowel syndrome. This review covers enzymes in the heme oxygenase pathway (HMOX, BVR, UGT1A1) that regulate bilirubin production and excretion, as well as the microbiome-driven breakdown of bilirubin into urobilinogen and its subsequent oxidation to urobilin. It highlights the inverse relationships among CRC, obesity, and inflammation and suggests that urobilin pathways influence CRC risk. Restoring bilirubin’s protective signaling and reducing circulating urobilin could open new avenues for prevention and treatment.

## Introduction

Approximately 2 million new cancer cases are expected annually, with breast, prostate, colon, and lung cancers constituting about half of these [1]. Colorectal cancer (CRC) continues to be a major global health concern, representing 10% of all cancer diagnoses, and is the third most common type, as well as the second leading cause of cancer death worldwide [2–5]. Risk factors for CRC include genetics, a sedentary lifestyle, obesity (body mass index [BMI] > 30 kg/m^2^) [6], and age over 50 years [7]. Alarmingly, obese individuals with a cancer diagnosis are more prone to develop a second primary cancer that is unrelated to their initial disease [8]. CRC is significantly affected by adiposity and increased body weight [9], which trigger a series of molecular mechanisms, including chronic low-grade inflammation, altered adipokine profiles, and insulin resistance [10]. Increased visceral fat releases inflammatory cytokines and promotes hyperinsulinemia, activating the insulin-like growth factor (IGF) axis and supporting tumor growth [10, 11]. Although much is known, the exact factors linking adiposity to CRC development remain to be elucidated.

Several mechanisms have been proposed to explain how obesity may lead to cancer. One involves the higher risk associated with increased body fat and weight, often linked to insulin resistance [6, 10, 11]. Another involves chronic inflammation, which promotes tumor growth [12]. A third concerns the dysregulation of adipokines—hormones produced by fat tissue that can either encourage or inhibit cell growth [13]. For example, leptin, an adipokine positively correlated with increased fat mass, is linked to abnormal cell proliferation [13], whereas adiponectin, which typically protects against tumor growth, is decreased in obesity [14], potentially allowing cancer to develop. Obesity also causes metabolic reprogramming [15], with disrupted metabolic hormones playing a key role in tumor development [16, 17]. A well-known but not fully understood phenomenon in obesity is that plasma bilirubin levels tend to be lower than in people with a lower BMI [18].

**Figure 1:**
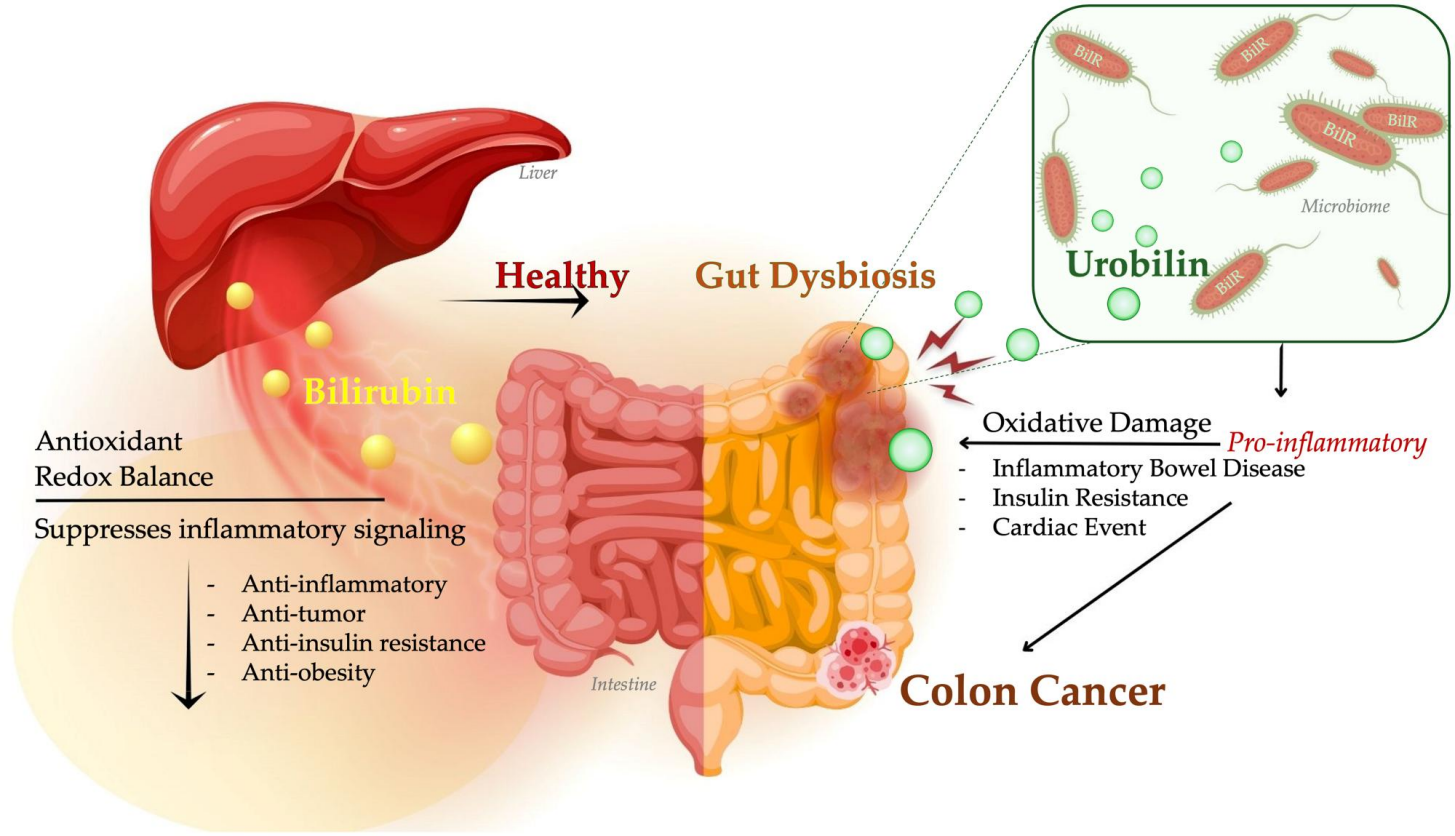
Hypothetical mechanisms that urobilin might contribute to colon cancer. Bilirubin within the liver–gut axis plays a crucial role in neutralizing reactive oxygen species (ROS), suppressing pro-inflammatory pathways, stimulating fat oxidation and utilization, enhancing insulin sensitivity, and mitigating the risk of tumor development. When the gut microbiome catabolizes bilirubin into urobilinogen, it rapidly oxidizes to urobilin, which may influence colorectal cancer risk and clinical outcomes. Elevated urobilin levels are generally observed in individuals with obesity and are even more pronounced in those afflicted with both obesity and insulin resistance. Although urobilinogen exhibits antioxidant properties, urobilin has been linked to DNA damage and metabolic disturbances, potentially facilitating colorectal carcinogenesis through mechanisms involving oxidative stress, insulin resistance, and inflammation.

Bilirubin functions as a hormone and an antioxidant, protecting cells from oxidative damage and inflammation [19, 20]. Its relationship with cancer is complex; generally, lower plasma bilirubin levels are associated with increased risk of certain cancers, while higher levels may offer protective effects [5]. This pattern is also observed in obesity, where higher BMI and insulin resistance correlate with lower plasma bilirubin levels [18]. Conversely, in some cancers, such as hepatocellular carcinoma (HCC), bilirubin levels are often significantly elevated [21]. An emerging hypothesis suggests that hyper-catabolism of bilirubin into urobilinogen, which is rapidly oxidized to urobilin [22], may play a crucial role in the onset of CRC (Fig. 1).

This work examines the heme oxygenase (HO) pathway, bilirubin production and breakdown, urobilin formation, and the impact of elevated plasma levels of bilirubin and urobilin, particularly their association with CRC. However, our understanding of how cancer influences bilirubin metabolism is still limited, particularly regarding whether bilirubin and its byproducts are harmful in cancer, especially CRC, and the role of the microbiome in this process. The discussion includes bilirubin generation, the mechanisms involved, the enzymes involved, and how the gut microbiota may contribute to CRC development.

## Bilirubin: generation and catabolism

### Bilirubin production and its excretion

**Figure 2:**
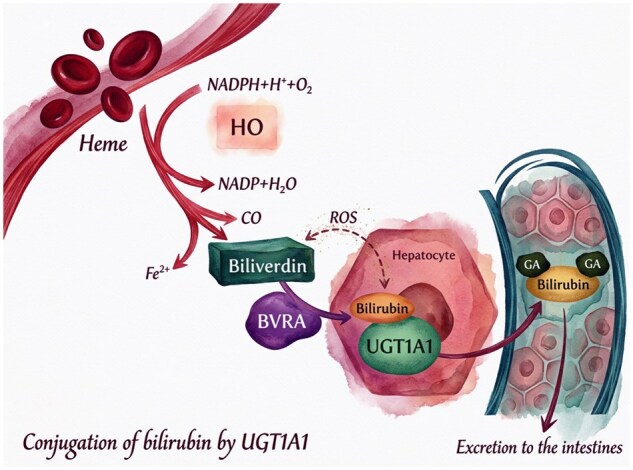
The production and clearance of bilirubin. Heme is degraded by heme oxygenase (HO) to biliverdin, carbon monoxide, and iron. Subsequently, biliverdin is reduced to bilirubin by biliverdin reductase A (BVRA). In hepatocytes, bilirubin undergoes conjugation by UGT1A1 and is subsequently excreted into bile for transport to the intestine. This pathway plays a crucial role in regulating systemic bilirubin levels and maintaining homeostasis.

Bilirubin production primarily originates in the spleen, arising from the breakdown of red blood cells via heme degradation by HO enzymes (*HMOX* gene) [23]. The rate-limiting step involves HO isozymes cleaving the heme/porphyrin ring to produce biliverdin, carbon monoxide, and ferrous iron (Fe^2+^) (Fig. 2) [24]. The primary isoforms are HO-1 (*HMOX1*), an inducible enzyme highly expressed in many tissues, especially those associated with erythrocyte and immune functions, such as bone marrow, spleen, and liver, and HO-2 (*HMOX2*), a constitutive form primarily found in the brain and testis [24, 25]. Biliverdin is reduced by biliverdin reductase (BVR) isozymes to form unconjugated bilirubin. The BVRA isozyme mainly produces bilirubin IXα [26], the predominant form in adults [24, 27]; the BVRB isozyme reduces biliverdin IXβ to bilirubin IXβ primarily during infancy [27]. When unconjugated bilirubin enters the bloodstream, it is bound to albumin to travel to the liver and become water-soluble through conjugation [23]. In the liver, bilirubin is conjugated by UDP-glucuronosyltransferase 1A1 (UGT1A1), enabling its transport into bile for intestinal excretion [18, 23, 24].

### Generation of urobilin

**Figure 3:**
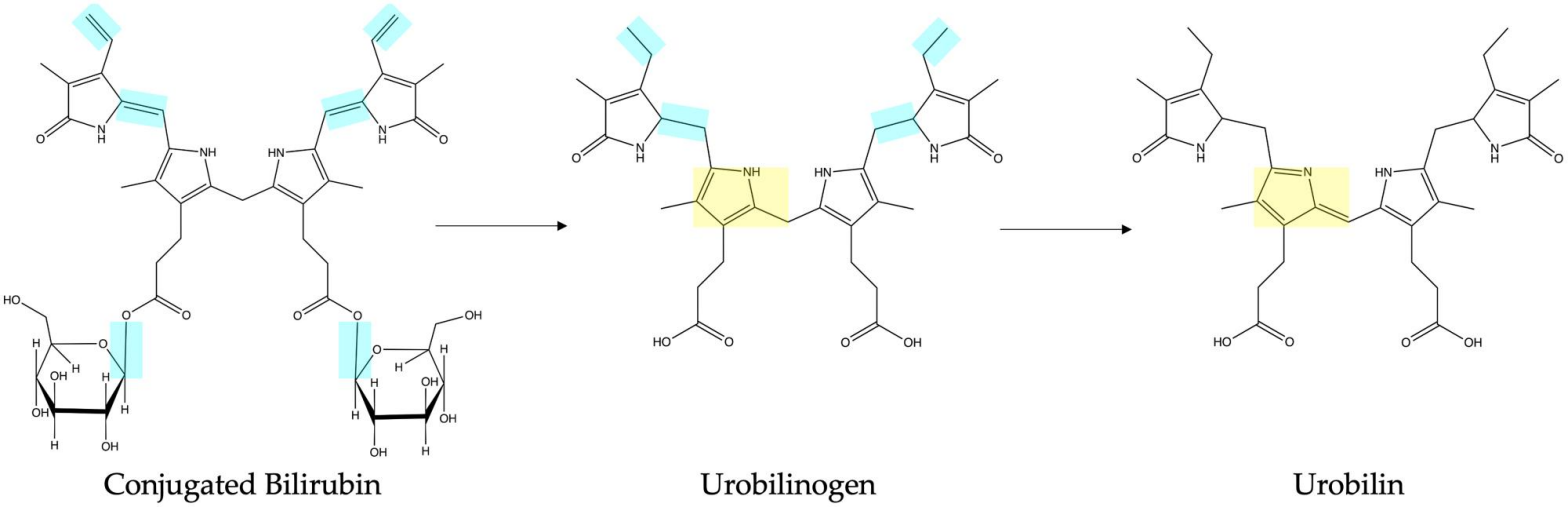
Structural alterations in the production of urobilin. In the colon, conjugated bilirubin is converted to urobilinogen by the bilirubin reductase (*BilR*) and β-glucuronidases (GUS), which are present in the gut microbiota. The BilR protein reduces the double bonds, while GUS removes the glucuronic acid molecule from conjugated bilirubin. Urobilinogen is then further oxidized to urobilin/stercobilin, which is reabsorbed or further metabolized and excreted through feces. *Yellow highlights show the differences between the chemical structures of urobilinogen and urobilin, while cyan highlights indicate the difference between urobilinogen and conjugated bilirubin.

Bilirubin metabolism in the gastrointestinal tract occurs in three main stages: deconjugation, reduction to urobilinogen, and further reduction to stercobilinogen (described further in [22, 28, 29]). As shown in Fig. 3, these processes begin with deconjugation, in which two glucuronic acid groups are removed from bilirubin. This enzymatic step is regulated by β-glucuronidase (GUS), which is expressed in the host or gut microbiota [30, 31] and is common among gut microbes [30]. The reduction of bilirubin to urobilinogen is essential for urobilin production, with gut microbes producing bilirubin reductase (BilR*)* enzyme, which reduces four bonds in bilirubin to generate urobilinogen [32]. Urobilinogen then spontaneously oxidizes to urobilin. Studying the BilR protein could help identify the bacterial species involved and reveal new targets for CRC treatments, as elaborated later.

## Bilirubin: physiological and signaling functions

### Bilirubin and oxidative stress

Bilirubin, historically regarded as a terminal waste product of heme catabolism, is now recognized as a biologically active antioxidant and cardiometabolic signaling molecule [19]. While abnormal levels may exert cytotoxic effects, physiologic and mildly elevated concentrations, 18–58 μM, confer cytoprotective, antioxidant, and anti-inflammatory properties [33, 34]. At physiologic levels, bilirubin functions as a potent lipophilic antioxidant, scavenging reactive oxygen species (ROS) and limiting lipid peroxidation [35]. Excessive ROS production is a major driver of colorectal carcinogenesis, contributing to oxidative DNA damage, genomic instability, and activation of oncogenic signaling pathways [5, 36–38]. By reducing oxidative stress, bilirubin attenuates early mutagenic events that promote colon tumor initiation [39]. Mildly elevated levels of bilirubin exert its antioxidant, anti-inflammatory, and signaling modulatory functions, reducing oxidative DNA damage, suppressing nuclear factor kappa-light-chain-enhancer of activated B cells (NF-κB) and signal transducer and activator of transcription 3 (STAT3) activation, preserving TP53 tumor suppressor activity, and inhibiting proliferative signaling pathways involved in colorectal tumorigenesis [35].

### Bilirubin and inflammation

Breast, colon, stomach, and pancreatic cancer progression is closely related to chronic inflammatory disease due to lipid accumulation and adiposity [40]. Beyond ROS scavenging, bilirubin exerts immunomodulatory effects by regulating key pro-inflammatory pathways [41] implicated in obesity-associated CRC. Bilirubin suppresses NF-κB activation, in part by reducing ROS-mediated degradation of inhibitor of kappa B (IκB) and limiting nuclear translocation of RELA/p65 [34, 37]. Because NF-κB drives transcription of genes, such as tumor necrosis factor (*TNF*), interleukin-6 (*IL6*), cyclooxygenase-2 (*COX2*), B-cell lymphoma 2 (*BCL2*), and cyclin D1 (*CCND1*), its inhibition reduces inflammatory signaling, tumor cell proliferation, and resistance to apoptosis in colon epithelial cells [42].

Bilirubin and its precursor biliverdin also influence cell cycle regulation. In a study examining the effects of bilirubin and biliverdin on the vascular smooth muscle cell (VSMC) cell cycle, it was observed that these treatments reduced phosphorylation of p38 mitogen-activated protein kinase (MAPK) [43], a kinase known to regulate cellular stress and survival [44]. Biliverdin has been shown to suppress NF-κB activity and induce cell cycle arrest by modulating cyclin-dependent kinases (CDKs), thereby limiting uncontrolled proliferation [36]. Additionally, bilirubin has been reported to influence tumor suppressor pathways by stabilizing or enhancing TP53 activity under oxidative stress conditions [45]. Preservation of p53 function promotes p21 transcription, leading to cell proliferation, cycle arrest, and inhibition of tumor cell proliferation [46].

### Bilirubin and its cardiometabolic effects

Obesity is a complex metabolic disorder characterized by excessive fat accumulation across multiple tissues. Its prevalence has risen markedly, with over 40% of adults in the United States affected [47]. Adiposity is linked to higher risks of non-communicable diseases [48], including cardiovascular disease (CVD), type 2 diabetes [49], and metabolic-associated steatotic liver disease (MASLD) [50], as well as several cancers [51]. In clinical settings, visceral fat (abdominal obesity) is a stronger predictor of CRC risk than overall obesity [52]. Weight loss, particularly through lifestyle changes or bariatric surgery, has been shown to reduce CRC incidence by approximately 27% in long-term studies [53, 54], and some reports found a 54% reduction in CRC risk among morbidly obese patients who underwent bariatric surgery [55]. Despite this strong link, current CRC treatment protocols do not account for patient weight, even though obesity may influence drug metabolism and immune response. People with obesity typically have reduced plasma bilirubin levels but elevated liver biomarkers, such as aspartate aminotransferase (AST) and alanine aminotransferase (ALT) [56]. Kipp *et al.* showed that plasma bilirubin levels in more than 164,000 patients were directly correlated with BMI, peaking at BMI 25 kg/m^2^, declining significantly after the overweight threshold of 30 kg/m^2^, and dropping sharply at severely obese BMIs of 40–50 kg/m^2^ [22]. In another study, Kipp *et al.* found that this pattern was exacerbated by insulin resistance, as measured by Homeostasis Model Assessment of Insulin Resistance (HOMA-IR), and that urobilin levels were also significantly higher [18]. CVD remains the leading cause of death worldwide among both men and women, especially in obese individuals with insulin resistance [57, 58]. Research indicates that bilirubin nanoparticles show potential in treating CVD [59], reducing body weight in experimentally obese animals, and enhancing insulin sensitivity [60–63]. The therapeutic uses of bilirubin have been considered [64].

Bilirubin acts as a hormone by binding directly to the nuclear receptor peroxisome proliferator-activated receptor alpha (PPARα) [33, 60, 62, 63, 65–67], inducing coregulator binding to PPARα [63, 68], thereby stimulating β-oxidation gene pathways [33, 67, 69–71]. A key finding of Kipp *et al.* was that urobilin levels were significantly higher in individuals with greater insulin resistance, indicating more bilirubin breakdown in those with more adiposity [22]. Conversely, fasting and caloric restriction increase bilirubin levels [72]; exercise raises plasma bilirubin [41, 73, 74], and, importantly, bilirubin levels normalize in response to weight loss [75]. Fasting slows gastric emptying, thereby reducing bilirubin excretion and enhancing enterohepatic circulation [76], and has also been associated with enhanced hepatic HO activity, leading to augmented bilirubin synthesis [77]. Bilirubin may play a role in the response to weight loss therapies; a retrospective study of 897 patients in a lifestyle-based weight management program reported bilirubin as a predictor of weight loss, with levels at or above 1.2 mg/dL more than twice as likely to be associated with weight loss of greater than 7% of body weight, as compared to those without elevated bilirubin levels [78].

Obesity-related cancers often involve disrupted lipid metabolism and chronic inflammation; bilirubin influences these processes through interactions with PPARα and its antioxidant properties. When bilirubin binds to PPARα, coregulators bind [68], eliciting gene responses that significantly affect lipid metabolism, decrease inflammatory gene activity, and enhance fatty acid oxidation [18, 79]. The activation of PPARα by bilirubin establishes a vital link between improved metabolic health and reduced CRC risk, with bilirubin nanoparticles showing potential as a therapy for CRC. Nonetheless, further research is required to confirm these observations.

### Bilirubin and colorectal cancer

Anatomically, the colon is divided into the right colon, comprising the cecum, ascending colon, and proximal two-thirds of the transverse colon, which primarily functions in water and nutrient absorption [3], and the left colon, consisting of the distal one-third of the transverse colon, descending colon, and sigmoid colon, which is primarily responsible for fecal storage and excretion [3]. While the etiology of CRC is determined by a multitude of factors that are not fully understood, genetic predisposition, dietary habits, exposure to carcinogens, and sedentary lifestyle all contribute to disease development in addition to obesity [3, 80]. Interestingly, polymorphisms in the *UGT1A1* gene, which influence bilirubin conjugation, predispose certain individuals to CRC [5].

Khoei *et al.* suggest that bilirubin plays a protective role in colorectal carcinogenesis [81]. Low baseline serum bilirubin levels have been consistently associated with higher CRC incidence rates and may reduce antioxidant capacity [4, 5]. Oxidative stress and chronic inflammation are major contributors to the pathogenesis of CRC, as well as several other cancers [82]. Bilirubin has demonstrated antioxidant and cytoprotective properties in both clinical and experimental studies [19]. Bilirubin is being investigated as a potential therapeutic or preventive agent, including in nanoparticle formulations designed to modulate oxidative stress in CRC [4, 5]. Clinical studies further support the protective role of bilirubin in CRC. In a cohort of stage IV CRC patients, pre-treatment levels of total bilirubin (TBIL), direct bilirubin (DBIL), and indirect bilirubin (IBIL) were measured and correlated with overall survival [4]. Higher IBIL levels were associated with improved survival, suggesting a potential protective effect [4]. The study further indicated that IBIL may induce apoptosis in colon cancer cells, possibly through mitochondrial depolarization, demonstrating a direct cytotoxic effect on tumor cells [4].

## Gut microbiota and colon cancer risks

### Effect of bilirubin reductase-containing bacteria on cancer progression

Specific species in the gut microbiome are responsible for the catabolism of conjugated bilirubin to urobilinogen [32]. Most of these species belong to the *Clostridium* genus within the Firmicutes Phylum, which possess BilR and GUS [32]. Bacterial bilirubin metabolism might serve two roles: utilizing glucuronic acid as a carbon source [31] or using bilirubin as an electron sink, rather than primarily as a carbon source. BilR carries out a double bond reduction of conjugated bilirubin, while GUS carries out its deconjugation by removing glucuronic acid (Fig. 3) [24]. Although BilR is predominantly found in the Firmicutes Phylum and *Clostridium* genus of the gut microbiota, GUS is expressed in four other gut phyla and human cells [24]. Members of this phylum and genus have shown an established relationship with cancer and inflammatory bowel disease [83–85]. *Clostridium symbiosum* levels are elevated in patients with CRC, particularly in tumor tissues, and correlate with an increased risk of colorectal adenoma recurrence [86]. Although outside of the *Clostridium* genus, *Ruminococcus gnavus* expresses BilR and can produce urobilin [32]. *Ruminococcus gnavus* has been positively associated with pro-inflammatory diseases, including Crohn’s disease, ulcerative colitis (UC), inflammatory bowel disease, obesity, type 2 diabetes, and CRC [87]. Obesity is associated with gut microbiota dysbiosis, often marked by an increased Firmicutes/Bacteroidetes ratio [88]. This underscores the potential importance of urobilin in the relationship among obesity, inflammation, and CRC. However, further investigations are necessary to ascertain whether urobilin is responsible for the association between urobilin-producing bacteria and disease pathogenesis.

### *Clostridium difficile* and its association with colon cancer

The *Clostridium difficile* toxin TcdB is linked to the activation of Wnt signaling, the generation of ROS, and a mucosal immune response [85]. *Clostridium difficile* is classified within the Firmicutes phylum and is linked to a heightened risk of colon cancer [89]. Microorganisms, such as Pks+ *E. coli* and *Clostridium difficile*, have evolved the ability to produce toxins, including TcdA, TcdB, and colibactin, which enhance their survival [85]. *Clostridium difficile* also expresses BilR and can produce urobilin, which may function analogously to other toxins produced by the bacterium. These toxins frequently cause damage to DNA and can initiate tumor-promoting processes in adjacent cells [85, 90]. *E. coli* has been shown to boost the conversion of bilirubin to urobilinogen by *Clostridium* bacteria in rats [91]. In germ-free rats infected with a single *Clostridium strain*, *E. coli* increased urobilin output in feces, although levels did not reach those observed in conventional mice [91]. Pks+ *E. coli* produces colibactin, a toxin linked to CRC [85]. Carcinogenesis may occur if DNA damaged by colibactin evades repair and proliferates. Moreover, Arthur *et al.* found that Pks+ *E. coli* was significantly more prevalent in rats with inflammatory bowel disease and CRC. The abundance of *Clostridium difficile* within the intestine is regulated by various factors, including diet and obesity. Patients with obesity are at an increased risk of *Clostridium difficile* infection [92]. Diets high in fat and sucrose are associated with *Clostridium difficile* infection and mortality [93, 94]. Interestingly, urobilin levels are lower in humans following healthy dietary patterns [95]. Future studies are necessary to determine whether urobilin contributes to the adverse effects of *Clostridium difficile* in CRC and to elucidate its role in the association between obesity and *Clostridium difficile*.

### *Clostridium septicum* and potential colon cancer risks

*Clostridium septicum* is a gram-positive, anaerobic, rod-shaped bacterium that exists as part of the normal human flora [84]. However, infection with *Clostridium septicum* can become dangerous and manifest as spontaneous gas gangrene, which is marked by rapid tissue death [96]. An association has been noted between gas gangrene, malignancies, and immunosuppression [84]. In one study, all seven patients with gas gangrene developed either CRC, blood cancer, or leukemia, and all patients with *Clostridium septicum* infection had a malignancy or immunosuppression [83]. Besides causing gas gangrene, *Clostridium septicum* also shows a positive link with CRC [84]. This may be because its anaerobic nature allows it to thrive in the low-oxygen environment created by gut tumors. However, it remains unclear whether the link between *Clostridium septicum* and colon cancer is purely opportunistic or causative, and whether *Clostridium septicum* has the capacity to generate urobilin.

### Effect of bile acid-reducing and short-chain fatty acid-producing bacteria on cancer progression

Certain gut bacteria play a protective role by modulating the immune system and producing anti-inflammatory and anticarcinogenic compounds [97]. Some bacteria generate short-chain fatty acids (SCFAs), such as butyrate, which help regulate intestinal pH and glycogenesis and create a hypoxic environment that prevents gut dysbiosis [97–99]. Butyrate is predominantly synthesized by *Faecalibacterium prausnitzii*, *Eubacterium rectale*, and *Eubacterium hallii* [100], and its production can be enhanced through increased dietary fiber consumption. Butyrate inhibits CRC tumor development and functions as a predictive biomarker for therapeutic response [101]. Gut bacteria also produce the SCFA acetate, which affects bile acid metabolism and generates unconjugated bile acids that may serve as metabolic regulators [97]. In addition to regulating metabolism, acetate promotes apoptosis [102] and inhibits growth [103] in colon cancer cells. Species, such as *Lactobacillus reuteri*, produce interleukin-10 via T-reg cells, supporting gut balance and reducing excessive inflammation [99]. Fecal levels of secondary bile acids correlate with mucosal and metabolic markers of CRC risk in both high- and low-risk adults and can be altered within a few weeks through dietary changes [104].

## Urobilin as a driver of colon cancer

**Figure 4:**
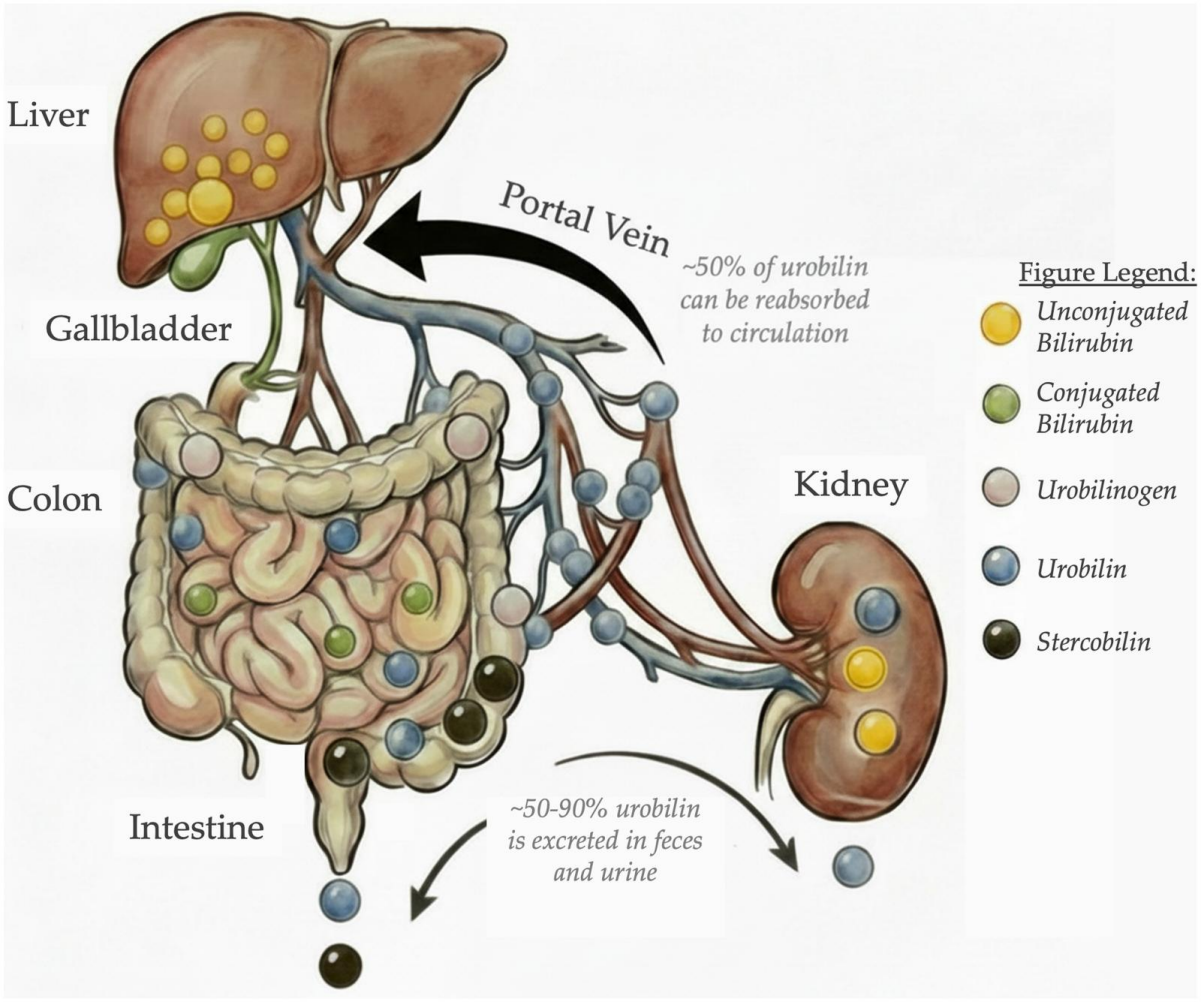
Unconjugated bilirubin catabolism to urobilin and reabsorption via hepatic vein. After unconjugated bilirubin is conjugated in the liver, it is converted to urobilinogen in the colon by the microbiome, which is then rapidly oxidized to urobilin. Urobilin is further oxidized to stercobilin, which is excreted and imparts color to the feces. Up to 50% of urobilin has been reported to be reabsorbed into the bloodstream via the hepatic portal vein. Some of the urobilin in circulation is transported to the kidneys for excretion in the urine.

As described before, urobilinogen is generated by the gut microbiota and oxidized to urobilin and stercobilin. Approximately 50% of urobilin is reabsorbed into circulation via the hepatic vein, with the remainder excreted through feces (Fig. 4) [22]. Nakamura *et al.* demonstrated that urobilinogen has weaker 2,2-diphenyl-1-picrylhydrazyl (DPPH) scavenging activity than bilirubin, tocopherol, and β-carotene [105]. However, when urobilinogen is converted to urobilin, it lacks antioxidant properties [106] and is associated with elevated inflammation [107] and insulin resistance [18]. Most studies have linked urobilinogen/urobilin to inflammatory signaling [107], which is activated by oxidative stress [49].

Although its precursor compounds have well-recognized functions, urobilin has been shown to have limited physiological signaling mechanisms. Urobilin has been demonstrated to bind to albumin and compete with bilirubin for albumin binding [108]. The association between urobilin and albumin is corroborated by a positive correlation with its autofluorescence intensity, which arises from the interaction between urobilin and albumin that induces fluorescence when they are bound [108]. Additionally, in silico docking confirmed the urobilin-albumin binding [108]. This interaction could be essential for transporting urobilin through systemic circulation. Currently, no additional binding proteins for urobilin have been identified, and its downstream signaling mechanisms remain unknown. Urobilin and stercobilin exhibited DNA-damaging effects in vitro in a study utilizing human cell lines HepG2 and Caco2 [90]. Cells exposed to intestinal tetrapyrroles (urobilin and stercobilin) showed significant comet formation and apoptosis in both cell lines [90]. Such effects may pose a cancer risk if DNA-damaged cells fail to undergo apoptosis and continue dividing. Future research is essential to elucidate the mechanisms underlying DNA damage induced by urobilin and stercobilin, as well as to clarify the role of urobilin under physiological conditions in the intestine and in CRC.

In addition to its DNA-damaging properties, urobilin could potentially act as a biomarker for cancer and other associated illnesses. A fecal metabolomic survey of 85 patients with CRC revealed elevated levels of bile acids, indoles, and urobilin [109]. Another study showed that fecal urobilinogen levels were increased in populations at high risk of CRC [110]. Urobilin is also an important biomarker for inflammatory bowel disease, obesity, and insulin-resistant type 2 diabetes (Fig. 1) [111], which are all risk factors for cancer. Research has shown that patients with Crohn’s disease have a disproportionate sphingolipid-to-L-urobilin ratio in feces, likely due to gut dysbiosis [111]. Faye *et al*. showed that patients with inflammatory bowel disease had an increased risk of intestinal malignancies due to long-term immunosuppressive drug exposure and chronic inflammation [112].

## Targeting mechanisms of bioconversion of bilirubin to urobilin

Bilirubin has been known to be converted to urobilinogen for over a century [22], and, when oxidized, forms urobilin [113], but the process remained unclear. In 2024, Hall *et al.* showed that bilirubin in the intestines is reduced by the BilR bacterial enzyme to form urobilinogen [32]. They also showed that certain bacterial subgroups, including the Firmicutes, had the potential to produce urobilinogen, and that when the reductase was isolated and transformed into *E. coli*, a non-bilirubin-reducing species, there was activity validating the discovery of the BilR enzyme [32]. This provides insight into how gut microbiome diversity affects urobilin production. This study further showed that patients with neonatal jaundice who also lack enzymes for bilirubin conjugation also lack BilR. Default-Thompson *et al.* described the evolution and lineages of the BilR gene, with a greater propensity to be found in the large intestines across various species [114].

Urobilinogen levels were shown to be influenced by nutritional status, as reported by Preidis *et al.,* who found that undernourished mice had significantly elevated plasma and urinary urobilinogen levels compared with control mice [115]. Zhao *et al*.'s work on bacterial advancements for re-establishing a healthy gut microbiome via bacterial probiotic supplementation in mice showed improved recovery from pathologic colitis but revealed an overall decrease in serum L-urobilinogen levels in treated mice [116]. In humans, urobilin was the sole plasma metabolite observed to significantly decrease with health-conscious dietary patterns [95], indicating a correlation with dietary intake and CRC, and suggesting a potential method to reduce the risk of diseases associated with elevated urobilin levels.

Three potential methods for altering bilirubin breakdown include inhibiting bilirubin excretion via UGT1A1, altering the gut microbiome, and directly targeting BilR. Utilizing a liver-specific UGT1A1 RNAi, Bates *et al.* reported an increase in plasma bilirubin and a decrease in urobilin, indicating the therapeutic potential of targeting UGT1A1 [117]. Hall *et al.* describe differences in urobilinogen production in healthy individuals versus those with irritable bowel syndrome (IBS) [32]. It is unclear whether these differences in expression are linked to natural inflammatory processes associated with IBS or to treatment-induced changes in the gut microbiome. In Crohn’s disease, urobilinogen levels were elevated at baseline compared with controls, whereas patients with UC had lower urobilinogen levels [118]. UC generally affects a greater portion of the large bowel. Regulatory effects on bacterial microenvironments and subsequent by-product generation, including urobilinogen, are much more specific. Little progress has been reported in targeting urobilinogen production, despite recent advances.

A prospective novel therapeutic approach focusing on the bioconversion of bilirubin to urobilin would target the bacterial species responsible for urobilin production or directly inhibit the BilR enzyme. Although numerous studies have found a positive correlation between antibiotic usage and colon cancer risk, this association is dependent on the antibiotic class and varies depending on the location within the colorectal tract, with an inverse relationship with rectal cancer [119, 120]. This underscores the necessity to develop specific inhibitors for BilR and the bacterial species that express it. Future research and preclinical studies are needed to evaluate whether direct modulation of the microbiota’s bilirubin metabolism has clinical efficacy in the treatment of CRC.

## Heme oxygenase pathway signaling and its association with cancer

HO regulates heme levels by catalyzing heme degradation and scavenging iron in red blood cells, processes that are essential for cellular oxygen and electron transport [121]. HO-1 upregulation is associated with oxidative stress and inflammation and serves as a cell-protective mechanism [122, 123]. Beyond its enzymatic role, HO-1 influences gene transcription, possibly promoting tumor growth and survival [122], and is often overexpressed in cancers, such as HCC, prostate, and breast, thereby increasing cell survival, proliferation, and angiogenesis [122]. HO-1 upregulation acts as a double-edged sword, protecting cells but supporting tumor progression and resistance [122, 123]. However, it remains uncertain whether this support is attributable to bilirubin or to other HO-1-derived products, including carbon monoxide and ferrous iron, as cellular iron is critical for tumor cell proliferation and metabolism [124].

BVRA is a key enzyme in bilirubin production and also functions as a serine/threonine and tyrosine kinase that mediates interactions between signaling proteins [27], including components of the MAPK/ERK cascade [125]. BVR regulates redox homeostasis [73, 74, 126–128], which is crucial for cellular homeostasis, and cancer may promote tumor survival by suppressing oxidative stress [129, 130]. BVR modulates MAPK, PI3K, and insulin signaling pathways, which have been associated with redox regulation and tumor cell proliferation [129]. Dysregulation of MAPK pathways plays a significant role in the onset and progression of several cancers, as their signaling is hyperactive in more than 40% of cases [131]. MAPKs relay signals from external stimuli, such as growth factors, cytokines, and neurotransmitters, which play a central role in regulating cell growth, proliferation, differentiation, and survival [130, 132]. Overactivation and dysregulation of MAPK signaling are evident in cancer, driving tumor growth and progression [44].

UGT1A1, a member of the UGT enzyme family, is essential for glucuronidating both exogenous and endogenous compounds, including bilirubin and steroid hormones [133, 134]. Its role in bilirubin breakdown is to convert unconjugated bilirubin into a water-soluble form for excretion, completing heme degradation [135]. Disruption of bilirubin homeostasis can increase oxidative stress, especially when high unconjugated bilirubin levels coincide with nonfunctional UGT1A1, leading to extremely elevated bilirubin levels. People with the polymorphism of this gene face a higher cancer risk due to toxicity and altered responses to chemotherapy [134]. Transcription of *UGT1A* is sex-specifically regulated in the liver, jejunum, and colon [134]. In hepatic tissue, females exhibit higher UGT1A1 expression and greater enzymatic activity than males [136]. This sexual dichotomy is directly influenced by progesterone, as no direct regulation has been demonstrated by estrogen [137]. Hormonal regulation of *UGT1A1* leads to differential expression and activity of the enzyme between men and women, resulting in sex-dependent circulating bilirubin levels, with females exhibiting lower concentrations than males [138]. There was also a stronger correlation between lower serum bilirubin levels and a higher incidence of CRC in females compared to males [139]. GUS enzymes deconjugate estrogen glucuronides, which are then reabsorbed; disruption of this process leads to significant alterations in estrogen levels, contributing to estrogen-related diseases [140]. These differences may elucidate the sex-dependent variations as genetic variants in *UGT1A1* show sex-dependent associations with CRC risk, with similar patterns reported in pancreatic cancer [134].

## Conclusions

Given the dual relationship between bilirubin and urobilin, it is crucial to investigate whether elevated bilirubin levels are associated with increased urobilin levels and if urobilin serves as a negative prognostic indicator in CRC. Additionally, a positive correlation exists between insulin resistance in obese individuals and urobilin levels. There is clear evidence linking obesity with a higher risk of CRC, along with obesity-related insulin resistance, lower bilirubin levels, and potentially higher urobilin levels, although further research is necessary. Increased expression of key enzymes in the HO pathway, such as HO-BVR-UGT1A1, may not be advantageous, as their overexpression is associated with cancer. While bilirubin, the product, could have therapeutic benefits for CRC, more studies are required to confirm its health advantages. Despite associations between urobilin and various risk factors, additional data are needed to elucidate the physiological roles of urobilin and other bilirubin-catabolized metabolites. Future preclinical investigations employing rodent models of CRC, including azoxymethane/dextran sodium sulfate, genetic, and transplantation models, are essential to elucidate the causal relationship between urobilin and tumor development, as existing evidence remains correlative. Furthermore, additional research into the protective effects of modulating *BilR*-containing bacteria, as well as the previously outlined therapeutic strategies, would be advantageous. Since the gut microbiome is affected in obesity and CRC, influencing bilirubin catabolism could open new avenues for colon cancer treatments.
